# Structure, kinetic properties and biological function of mechanosensitive Piezo channels

**DOI:** 10.1186/s13578-020-00522-z

**Published:** 2021-01-09

**Authors:** Xiang-Zhi Fang, Ting Zhou, Ji-Qian Xu, Ya-Xin Wang, Miao-Miao Sun, Ya-Jun He, Shang-Wen Pan, Wei Xiong, Zhe-Kang Peng, Xue-Hui Gao, You Shang

**Affiliations:** 1 Department of Critical Care Medicine, Union Hospital, Tongji Medical College, Huazhong University of Science and Technology, Wuhan, China; 2 Institute of Anesthesiology and Critical Care Medicine, Union Hospital, Tongji Medical College, Huazhong University of Science and Technology, Wuhan, China

**Keywords:** Piezo, Mechanotransduction, Function, Ion channel

## Abstract

Mechanotransduction couples mechanical stimulation with ion flux, which is critical for normal biological processes involved in neuronal cell development, pain sensation, and red blood cell volume regulation. Although they are key mechanotransducers, mechanosensitive ion channels in mammals have remained difficult to identify. In 2010, Coste and colleagues revealed a novel family of mechanically activated cation channels in eukaryotes, consisting of Piezo1 and Piezo2 channels. These have been proposed as the long-sought-after mechanosensitive cation channels in mammals. Piezo1 and Piezo2 exhibit a unique propeller-shaped architecture and have been implicated in mechanotransduction in various critical processes, including touch sensation, balance, and cardiovascular regulation. Furthermore, several mutations in Piezo channels have been shown to cause multiple hereditary human disorders, such as autosomal recessive congenital lymphatic dysplasia. Notably, mutations that cause dehydrated hereditary xerocytosis alter the rate of Piezo channel inactivation, indicating the critical role of their kinetics in normal physiology. Given the importance of Piezo channels in understanding the mechanotransduction process, this review focuses on their structural details, kinetic properties and potential function as mechanosensors. We also briefly review the hereditary diseases caused by mutations in Piezo genes, which is key for understanding the function of these proteins.

## Introduction

Mechanotransduction, the process by which mechanical stimuli are converted into electrochemical signals, is essential for various biological processes, including neuronal cell development, pain sensation, and red blood cell volume regulation [1–3]. As pivotal mechanosensors of in the mechanotransduction process, mechanosensitive (MS) ion channels have been found in organisms from bacteria to mammals [4, 5]. Extensive studies have revealed a variety of ion channels in eukaryotic cells that are able to sense various forms of mechanical forces (Table 1). These ion channels include transient receptor potential (TRP) channels and voltage-gated Na^+^, K^+^ and Ca^2+^ channels, whose dysfunction may be associated with human genetic diseases [29]. Notably, the MS candidates identified in invertebrates either have no homologues (e.g., TRPN) or no functional conservation (e.g., DEG/ENaC/ASIC) in mammals [30, 31]. Furthermore, most MS candidates (the TRP channel in particular) are activated not only by mechanical stimuli by but also by chemicals, temperature, osmolarity, and heat (> 27–34 °C) [32]. Defining the molecular details of MS cation channels in mammals is therefore of paramount importance to understand the mechanotransduction process and find potentially novel therapeutic strategies for mechanosensitivity disorders.

**Table 1:** Mechanosensitive ion channels in eukaryotic

Channel family	Channel isoforms	Ref.
TRP channels	TRPA1	[6]
	TRPC1	[7]
	TRPC6	[8]
	TRPV1	[9]
	TRPV4	[10]
	TRPM4	[11]
	TRPM7	[12]
	TRPN	[13]
	TRPP2	[14]
K + channels	Shaker (Kv1.1)	[15]
	Ca^2+^-activated K^+^ (BK)	[16]
	TREK1/2	[17]
	TRAAK	[18]
	HCN2	[19]
Na^+^ channels	Nav1.5	[20]
Ca^2+^ channels	L-type	[21]
	N-type	[22]
	T-type	[23]
Cl^−^ channels	CFTR	[24]
OSCA protein family	*Sc*CSC1, *Hs*CSC1	[25]
DEG/ENaC superfamily	C.elegans MEC (MEC-4, MEC-10)	[26]
	ASIC	[27]
Other channels	TMC1/2	[28]

*TRP* transient receptor potential,* DEG/ENaC* Degenerin/epithelial sodium channel

In 2010, Coste et al. [33] revealed a novel family of mechanically activated (MA) cation channels in eukaryotes consisting of Piezo1 and Piezo2 channels, which have been proposed as the long-sought-after MS ion channels in mammals. The Piezo1 channel is present in nonsensory tissues, with particularly high expression in the lung, bladder, and skin; by contrast, the Piezo2 channel is predominantly present in sensory tissues, such as dorsal root ganglia (DRG) sensory neurons and Merkel cells [33]. Since their discovery, tremendous effort has been made to reveal the structures and biological functions of Piezo 1 and 2. The partial molecular structure of a Piezo channel was determined by cryo-electron microscopy (cryo-EM) [34–38]. Furthermore, Piezo channels have been linked to various pathological and physiological processes, including erythrocyte volume regulation [39], cell division [40], and innate immunity [41]. Moreover, Piezo channel mutations are associated with multiple hereditary human diseases, such as autosomal recessive congenital lymphatic dysplasia [42], hereditary xerocytosis [43] and an autosomal recessive syndrome of muscular atrophy with perinatal respiratory distress [44]. Considerable progress has been made towards characterizing the structural features, physiological significance, and biophysical properties of Piezo proteins. Given the importance of Piezo channels in understanding mechanotransduction processes, this review focuses on their structural details, kinetic properties and potential functions as mechanosensors. We also briefly review the hereditary diseases caused by mutations in the Piezo genes, which is key to understanding their functions.

## Structure of Piezo channels

**Fig. 1:**
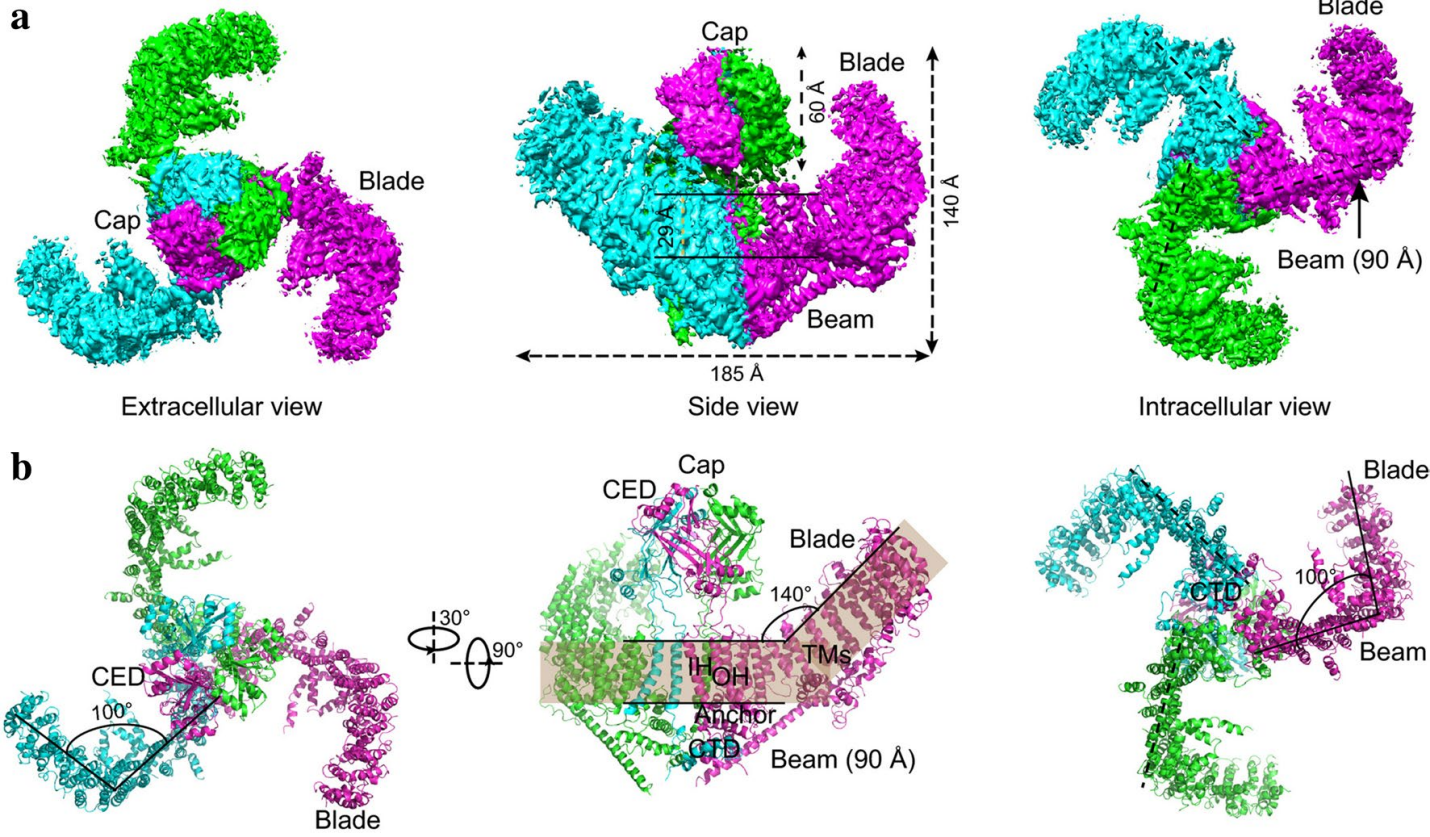
Cryo-EM structure of the mPiezo1 channel. (adapted from Zhao et al. [35]). **a** Multiple views of the sharpened map of the trimeric channel with the major domains labeled, with the three subunits colored red, green and blue. **b** Cartoon model in which the three subunits are colored red, green and blue. In the middle panel, the front subunit has been omitted to provide a better view of the curvature of the TMs

Piezo proteins have an uncommonly large predicted size of approximately 2500 amino acids and encompass numerous transmembrane (TM) regions [33]. Subsequent research has revealed that the mouse Piezo1 (mPiezo1) channel is an evolutionarily conserved pore-forming ion channel directly gated by membrane stretch [45, 46]. Several published cryo-EM studies have revealed that mPiezo1 exhibits a three-bladed, propeller-shaped homotrimeric structure that includes a central cap, three peripheral blade-like structures on the extracellular side, three long beams on the intracellular side that bridge the blades to the cap, and a TM region between these features [34–38] (Fig. 1).

## **Structure of the Piezo1 channel**

### Unprecedented 38-TM topology

**Fig. 2:**
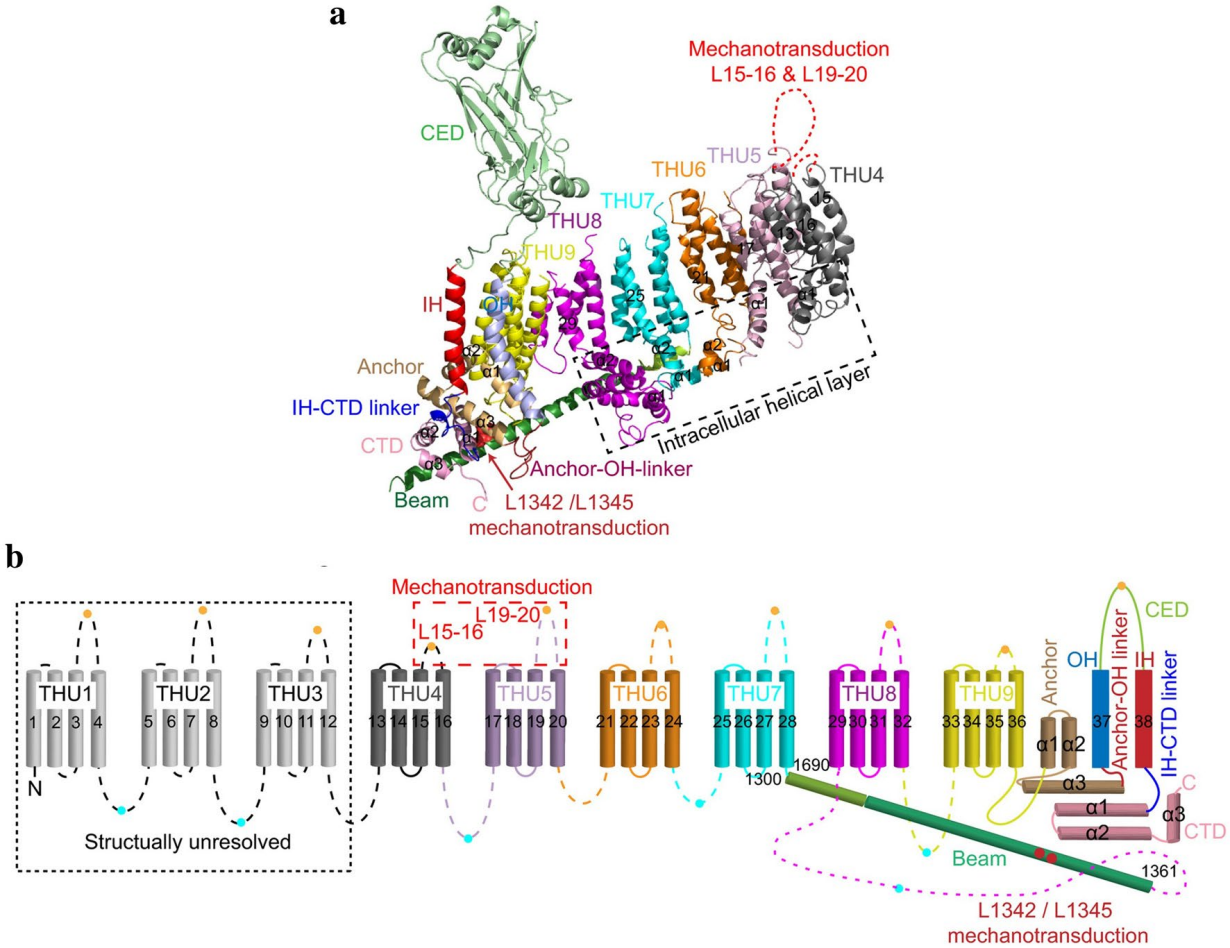
A 38-TM topology model and key functional sites in mPiezo1. (adapted from Zhao et al. [35]). **a** A model showing one subunit with individual THUs and featured structural components. Residues L1342 and L1345 in the beam are indicated by red spheres. **b** A 38-TM topology model color-coded to match the cartoon model in A

Piezo channels are predicted to possess an unusually large number of TM regions, ranging from 10 to 40 [33, 45, 47]. Zhao et al. [35] recently produced high-resolution structures of mouse Piezo1 (mPiezo1), revealing a unique 38-TM topology in each subunit (Fig. 2a, b). The two TM regions (TM37 and TM38) closest to the center of the protein are designated as the inner helix (IH) and outer helix (OH), respectively, and enclose the transmembrane pore of the central pore module. The other 36 TM regions (TM1-36) form a curved blade-like structure with nine repetitive folds containing 4 TM regions each, named transmembrane helical units (THUs)

### Central cap

**Fig. 3:**
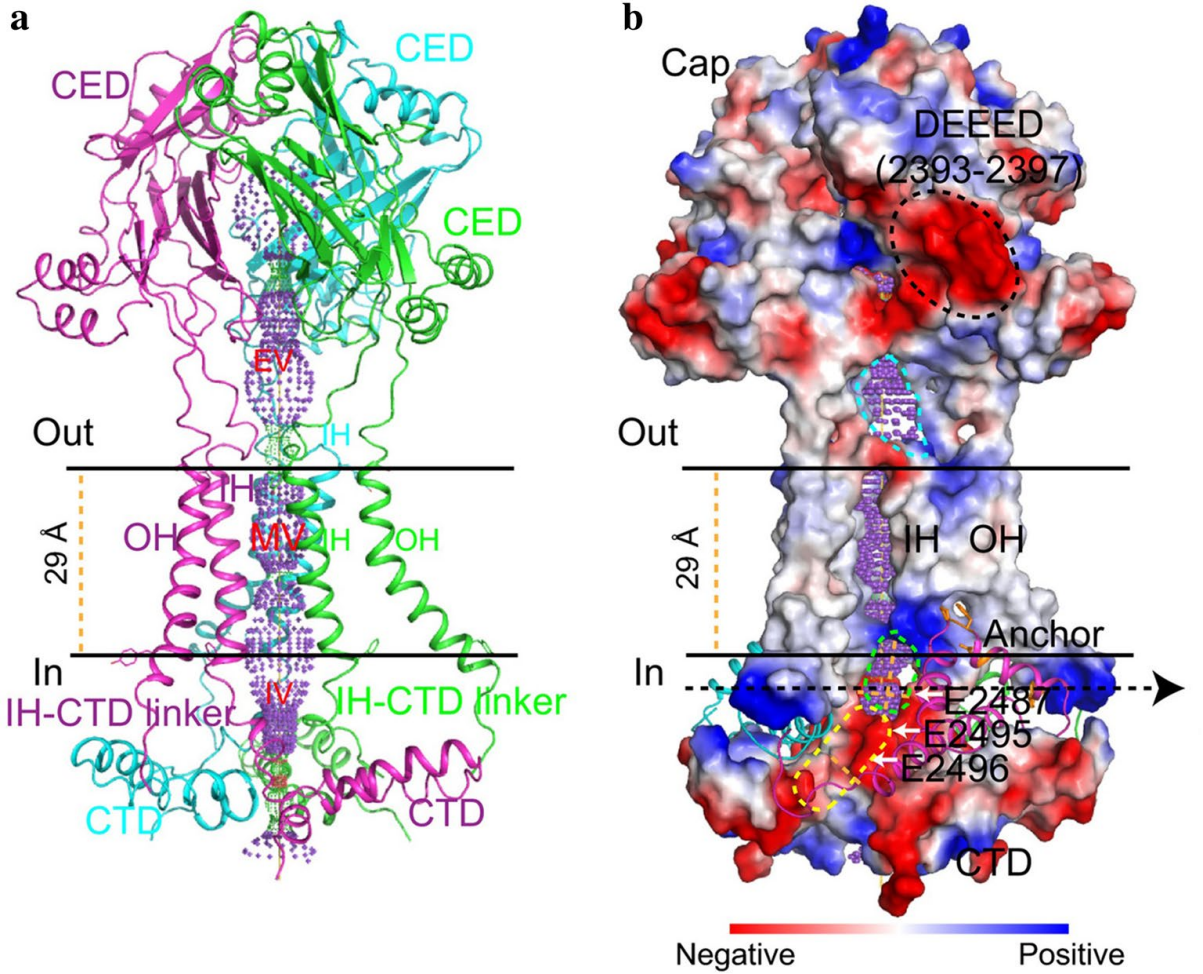
Structure of the central pore module. (adapted from Zhao et al. [35]). **a** Ribbon diagram of the ion-conduction pore comprising the OH, CED, IH, and CTD from three color-coded subunits. The central solvent-accessible pathway is marked with dotted mesh generated by the program HOLE. **b** Pore module presenting the surface electrostatic potentials showing negative (red) and positive (blue) potential. Extracellular and intracellular fenestrations are marked by cyan and green dashed lines, respectively. The lateral portal is marked by yellow dashed lines

Kamajaya and colleagues [48] employed topological prediction modeling and found that residues 2210 to 2457 in Piezo1 form an extracellular loop following the last TM region from the C-terminus, defined as the C-terminal extracellular domain (CED) **(**Fig. 1**)**. The deletion of residues 2218 to 2453 from the Piezo1 protein abolished expression of the central cap [34], suggesting that this region trimerizes to form the central cap (Figs. 1 and 3). Further analysis revealed that the central cap consists of the CED in the form of a trimeric complex that encloses an extracellular vestibule (EV) with openings [34, 49] (Fig. 3*)*.

### Anchor

A hairpin structure, referred to as the anchor, connects the OH-IH pair to the C-terminal domain (CTD) plane, which moves the OH-CED-IH-containing region of one subunit into the neighboring subunit in a clockwise direction (Figs. 1 and 2). The anchor is made up of three helices (α1, α2, and α3). Helices α1 and α2 were found to organize into an inverted V-shaped structure, which maintains the integrity of the ion-conducting pore (Fig. 2b). In parallel with the membrane plane, the long α3 helix links to the OH via a lysine-rich anchor-OH linker that interacts with the polar residue-rich α2–3 turn in the anchor and the glutamate-rich region of the CTD. A few mutations in Piezo1 at locations including KKKK (2182-K2185), T2143, T2142 (T2127 in human Piezo1), R2514, E2523, and E2522, which are located in α3 in the anchor, have been reported to cause severe disease [35, 50]. Additionally, SERCA2, a Piezo-interacting protein, suppresses Piezo1 by acting on the anchor-OH linker [51]. These findings support the structural and functional importance of the anchor region.

### The long intracellular beam

On the intracellular surface, Piezo1 contains three beam-like structures 90 nm in length that are organized at a 30° angle relative to the membrane plane (Figs. 1 and 2). Residues H1300-S1362 form the beam structure. The large intracellular THU7-8 loop, which contains approximately 390 residues, might provide the beam with the structural basis for mechanical transmission. Functionally, the three long intracellular beams not only support the whole TM skeleton but also physically bridge the distal THUs to the central ion-conducting pore. When residues 1280 to 1360 (which form this beam structure) were deleted, the resulting mutant protein was absent, suggesting the structural importance of the beam [35].

### Highly curved blades

The nine peripheral THUs in each subunit form blade-like structures, with each blade twisted clockwise (Fig. 1b). The proximal TM25–TM36 and peripheral TM13-24 interact at a 100° angle, as viewed from 90º relative to the plasma membrane plane, and a 140° angle, as viewed from a line parallel to the plasma membrane plane. Another important feature of the blades is the L-shaped helical structures formed by TM13, TM17, TM21, TM25 and TM29. Both identifiable structural features appear to be ideal for not only for mechanosensation but also for the induction of local membrane curvature. Intriguingly, the peripheral TM13-24 appears to be within a highly curved membrane plane, indicating that the Piezo1 channel can curve the membrane in which it resides. This is consistent with past studies implying that Piezo1 ion channels can be regulated by cellular membrane curvature and tension [46, 52, 53].

### The ion-conducting pathway

As pore-forming ion channels, Piezo proteins contain a trimeric ion-conducting channel made up of residues 2,189 to 2,547, which contain the last two TMs (Fig. 3). The continuous central channel consists of three parts, an EV within the cap region, a transmembrane vestibule (MV) within the membrane, and an intracellular vestibule (IV) underneath the membrane. Both the EV and IV possess an opening that connects to the MVs, which are positioned above and below the membrane. Importantly, DEEED (2393–2397), a patch of negatively charged residues residing in the opening of the extracellular “cap” structure consisting of the CED, is required to ensure efficient ion conduction and determine the selection of cations over anions. Additionally, two critical acidic residues, E2495 and E2496, located at the CTD-constituted IV, may be responsible for divalent calcium ion selectivity, unitary conductance and pore blockage.

## **Structure of the Piezo2 channel**

Similar to Piezo1 channels, Piezo2 channels are large membrane proteins consisting of over 2,800 residues. However, the Piezo2 channel and Piezo1 channel share approximately only 42% sequence homology [33]. Recent studies [38, 54] have shown that the overall structure of the Piezo2 channel is very similar to that of Piezo1 in that it forms a three-bladed, propeller-like homotrimeric structure comprising a central ion-conducting pore module and three peripheral blades with 38 TMs.

In the Piezo2 channel, the charged residues at the interface between the beam and the CTD are required to ensure the normal mechanosensitivity of the channel [54]. Moreover, single-channel recordings indicated that a previously unrecognized intrinsically disordered domain adjacent to the beam acts as a cytosolic plug that limits ion permeation, possibly by clogging the inner vestibule in both Piezo1 and Piezo2 [54]. Furthermore, by structurally comparing the Piezo1 and Piezo2 channels, Wang et al. found that the Piezo2 channel has additional constriction sites at L2743, F2754 and E2757 that might serve as a transmembrane gate controlled by the cap domain [38].

## Lever-like mechanotransduction mechanism

**Fig. 4:**
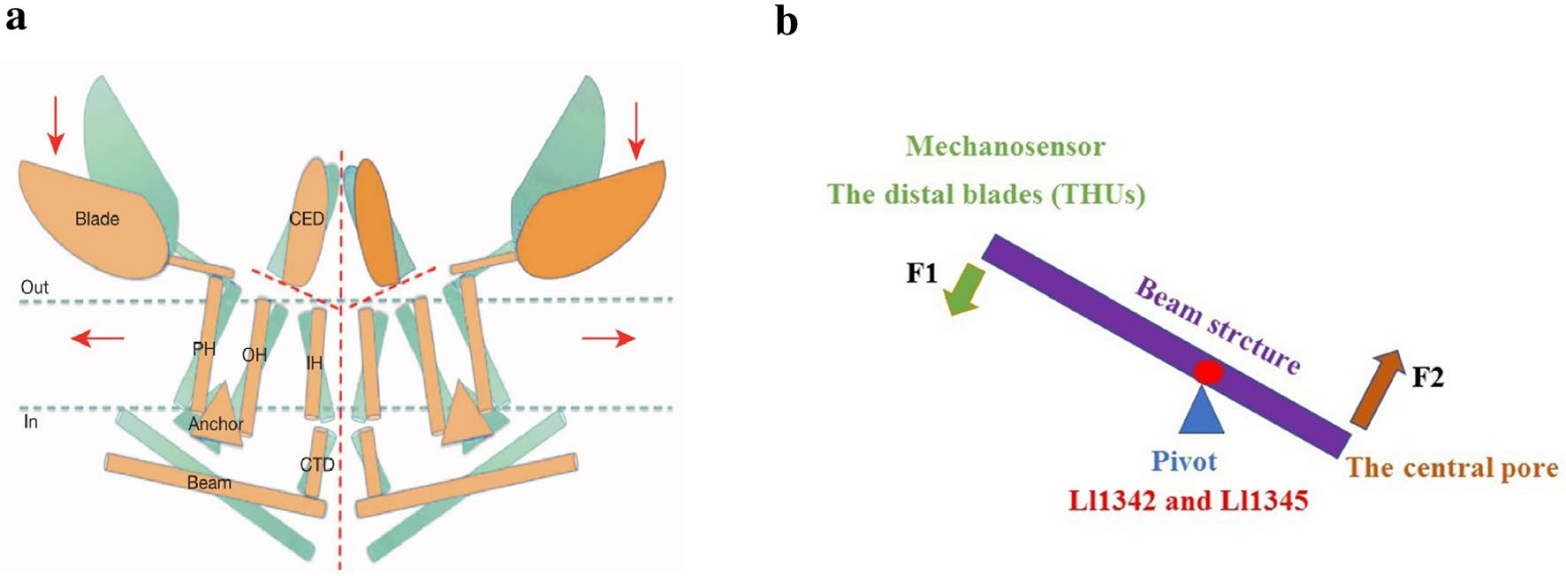
Model of the lever-like mechanotransduction model. The curved blades can act as a mechanosensor, while the beam structure, with residues Ll1342 and Ll1345 acting as a pivot, can act as a lever-like apparatus. Coupling of the distal blades and central pore through the lever-like apparatus converts mechanical force into cation conduction. **a** Proposed model of the force-induced gating of Piezo channels. The blue and orange models represent the channel in its closed and open states, respectively. Red dashed lines indicate possible ion-conduction pathways. Adapted from Ge et al. [34]. **b** A lever-like mechano-gating model in Piezo1. The blue and red dashed arrows indicate input and output forces, respectively

Based on the unique topological features of the mPiezo1 channel, a lever-like mechanotransduction mechanism to explain its extraordinary mechanosensitivity was proposed [34, 4] (Fig. 4). In the mPiezo1 channel, the curved blades composed of THUs can act as a mechanosensor, while the beam structure, with the residues Ll1342 and Ll1345 acting as a pivot, can act as a lever-like apparatus. Coupling the distal blades and central pore through the lever-like apparatus converts mechanical force into a force used for cation conduction.

Because the pivot of the lever is positioned closer to the central pore than to the distal blades, the input force is effectively amplified through the lever-like apparatus. Additionally, a large conformational change in the distal blades is converted into a relatively slight opening of the central pore, allowing cation-selective permeation.

## Kinetics properties of Piezo channels

### Activation mechanisms of Piezo channels

Normal Piezo channel kinetics can be modeled with three states: open, closed, and inactivated; these states have emerged, collectively, as an important mechanism in Piezo channel function [55]. Studies have proposed that the Piezo1 channel is gated directly by bilayer tension that can be modified by cytoskeletal proteins and linkages to the extracellular matrix (ECM) [46, 52, 53]. For example, in overhydrated red blood cells (RBCs), Piezo1-mediated Ca^2+^ influx activates K^+^ efflux through the Gardos channel (KCa3.1), which in turn leads to water loss and RBC dehydration [39].

**Fig. 5:**
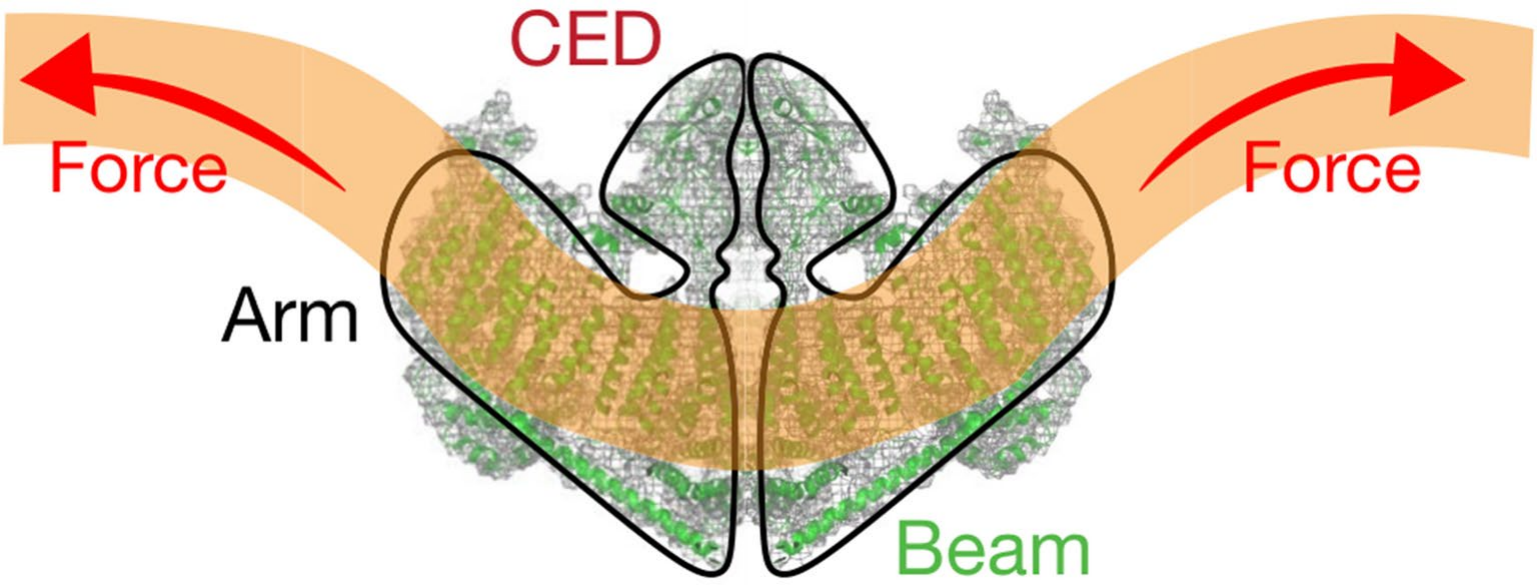
Model of the membrane doming mechanisms. Changes in membrane curvature lead to a gating force applied to the Piezo1 channel

Piezo1 and Piezo2 channels not only exhibit a three-bladed, propeller-shaped trimeric architecture but also have the ability to locally deform lipid membranes into a dome-like shape [35, 38]. In addition, changes in the projection area of Piezo channels from closed to open are essential for their mechanosensitivity; this was investigated by calculating the available free energy [36]. Based on these findings, the membrane dome mechanism was proposed and experimentally proved to explain the activation mechanisms of Piezo channels [36, 56] (Fig. 5). Essentially, the dome shape created by Piezo channels in their closed conformation acts as a potential energy source for MS gating. Under tension, lateral membrane tension flattens the Piezo dome, which increases the energy of the membrane-channel system in proportion to the expansion of the projected area of the dome. Piezo channels then open due to the relative energy difference. This mechanism can account for the highly sensitive mechanical gating of Piezo channels with a cation-selective pore. Although the membrane dome mechanism explains the exquisite mechanosensitivity of Piezo channels, it does not consider the shape of the surrounding membrane. Haselwandter et al. [57] proposed the membrane footprint hypothesis, which states that the Piezo1 channel deforms the shape of the membrane outside the perimeter of the channel such that it exhibits a curved membrane footprint, which amplifies the sensitivity of Piezo1 to changes in membrane tension. Nevertheless, further experiments are needed to test and refine these hypotheses.

### Inactivation kinetics of Piezo channels

Various types of mechanical stimulation trigger Piezo channel activation and sequentially elicit an MA current with rapid decay, even in the presence of continued stimulation, due to rapid inactivation [58]. Coste et al. [33] first described detailed information about the voltage-dependent inactivation kinetics of Piezo channels, characterized as fast at rather negative membrane potentials and slow at rather positive membrane potentials. Additionally, Piezo1 channel inactivation is relatively slow compared with Piezo2 channel inactivation. Several point mutations in Piezo channels have been reported to slow down the inactivation process [43, 59], which produces larger cation fluxes and results in various human diseases. Given its demonstrated key role in normal channel function, we next review what is known about the inactivation kinetics of Piezo channels with a focus on the inactivation mechanism.

The available information regarding the structures (residues/domains) and human disease-related point mutations have helped to clarify the mechanisms of ion channel inactivation. Currently, six gain-of-function mutations associated with dehydrated hereditary xerocytosis (DHS) have been found to slow the inactivation rate of Piezo channels (Table 2), most of which are clustered at the central core region of the Piezo channel structure. This implies that the pore region, which contains an OH, an IH, an extracellular cap domain and an intracellular CTD, determines the kinetics of inactivation. Further detailed links between structural domains and inactivation kinetics have been investigated. Wu et al. identified that the distinct inactivation kinetics of Piezo1 and Piezo2 channels and characteristic voltage-dependent inactivation appear to be determined by the C-terminal extracellular domains (cap domain) [67]. Two potential inactivation gates within the IH and CTD have been thought to be sufficient for the normal inactivation of the Piezo1 and Piezo2 channels [7, 68], . Recently, three small subdomains within the extracellular cap were shown to individually confer Piezo channel inactivation [69]. These results support the idea that the ion-conducting pore region of Piezo channels is essential for their inactivation properties.

**Table 2:** Mutations in Piezo1 and Piezo2 Associated with Human Diseases

Protein	Mutation in amino acids	Disease	Channel Domin	Functial phenotype	Reference	
Piezo1	A2003D	DHS	PH	Unrepored	[50]	
	G718S	DHS		Unrepored	[50]	
	G782S	DHS	PH	Unrepored	[50]	
	R808Q	DHS	PH	Unrepored	[50]	
	S1117L	DHS	PH	Unrepored	[50]	
	R2488Q	DHS	CTD	Unrepored	[50]	
	K2166–2169 del c	DHS	OH	Unrepored	[50]	
	A2020V	DHS	PH	Unrepored	[50]	
	M2225R	DHS	CED	Slowed inactivation	[43, 59]	
	T2127M	DHS	Anchor	Slowed inactivation	[50, 59]	
	R2456H	DHS	IH	Slowed inactivation	[43, 50, 59]	
	R1358P	DHS	PH	Slowed inactivation	[59]	
	A2020T	DHS	PH	Slowed inactivation	[59]	
	E2496ELE	DHS/HA	CTD	Slowed inactivation	[59]	
	H702Y	CAP		Unrepored	[60]	
	I1007M	CAP		Unrepored	[60]	
	V1712M	CAP		Unrepored	[60]	
	Y1763X	CAP		Unrepored	[60]	
	R1955C	CAP	PH	Unrepored	[60]	
	E1630X	GLD	PH	Unrepored	[61]	
	E755X	GLD	PH	Unrepored	[61]	
	L939M	GLD		Unrepored	[61]	
	Q2228X	GLD	CED	Unrepored	[61]	
	P2430L	GLD	IH	Unrepored	[61]	
	V2171F	GLD	Anchor-OH	Unrepored	[61]	
	R2456C	GLD	IH-CTD	Unrepored	[61]	
	F2458L	GLD	IH-CTD	Unrepored	[61]	
	G2029R	GLD		Unrepored	[62]	
	S1153Wfs21 splic donor	GLD	PH	Unrepored	[62]	
	G2029R	GLD		Unrepored	[62]	
Piezo2	M712I	DA5	PH	Unrepored	[63]	
	M712V	DA5	PH	Unrepored	[64]	
	M998T	DA5	PH	Unrepored	[64]	
	T2221I	DA5	PH	Unrepored	[64]	
	S2223L	DA5	PH	Unrepored	[64]	
	T2356M	DA5	Anchor	Unrepored	[64]	
	R2686H	DA3	IH-CTD	Unrepored	[64]	
	R2686C	GS/DA5	IH-CTD	Unrepored	[64]	
	R2718L	DA5	CTD	Unrepored	[64]	
	R2718P	DA5	CTD	Unrepored	[64]	
	Y2737Ifs7*	DA5	CTD	Unrepored	[64]	
	S2739P	DA5	CTD	Unrepored	[64]	
	W2746X	DA3	CTD	Unrepored	[64]	
	E2727del	DA5	CTD	Unrepored	[64, 65]	
	I802F	DA5	PH	Faster recovery from inactivation	[65]	
	A1486P	DA5	PH	Unrepored	[66]	

*CED * extracellular domain, *CTD* C-terminal extracellular domain, *PH* peripheral helices,* OH* outer helix,* IH* inner helix,* DA5,*distal arthrogryposis subtype 5, *DHS,* dehydrated hereditary stomatocytosis, *GLD* generalized lymphatic dysplasia, *GS* Gordon syndrome, *HA* hemolytic anemia

Interestingly, a slowly inactivating MS current in mouse embryonic stem cells (mESs) has been described that is dependent on the Piezo1 channel [70]. However, heterologous expression of Piezo1 cDNA from mES cells displays fast inactivation kinetics, indicating that additional regulatory mechanisms other than the amino acid sequence determine the slow kinetics of the Piezo1 channel in mES cells [70]. Recently, sphingomyelinase activity has been revealed to be a crucial determinant of Piezo1 inactivation [71]. Various modulators, such as pH, temperature, divalent ion concentrations, alternative splicing, osmotic swelling, membrane lipid composition, co-expression of other membrane proteins, and G-protein-coupled pathways have also been reported to regulate the Piezo channel kinetics [55, 72–79]; however, we still know very little about the relationships among these factors and pivotal structural domains.

### Pharmacological modulators of Piezo channels

Despite the relatively recent discovery of Piezos, there has been progress regarding small-molecule modulators of Piezo1. Piezo1 chemical activators, including Yoda1 and Jedi1/2, were able to open Piezo1 ion channels in the absence of mechanical stimulation. Jedi1/2, a novel hydrophilic Piezo1 chemical activator, acts through the peripheral blades and utilizes a peripheral lever-like apparatus consisting of the blades and a beam to gate the central ion-conducting pore [80], whereas Yoda1 acts as a molecular wedge, facilitating force-induced conformational changes, effectively lowering the channel’s mechanical threshold for activation [81]. However, the reason why Yoda1 does not efficiently activate the Piezo2 channel is unclear. Specific inhibitors of Piezo1 are in their infancy. As nonspecific inhibitors of the ion pore in stretch-activated ion channels, gadolinium and ruthenium red have also been shown to block mouse Piezo1 channels with IC50 values of approximately 5 mM [45]. The commonly used toxin inhibitor of mechanosensitive channels, GsMTx4, was also found to inhibit the Piezo1channel [82], but it might not bind Piezo1 directly, rather acting via modulating local membrane tension near the channel [83, 84]. Dooku1, an analog of Yoda1 without a stimulatory effect, antagonizes Yoda1-evoked activation of Piezo1 and aortic relaxation [85].

## Function of Piezo channels

**Fig. 6:**
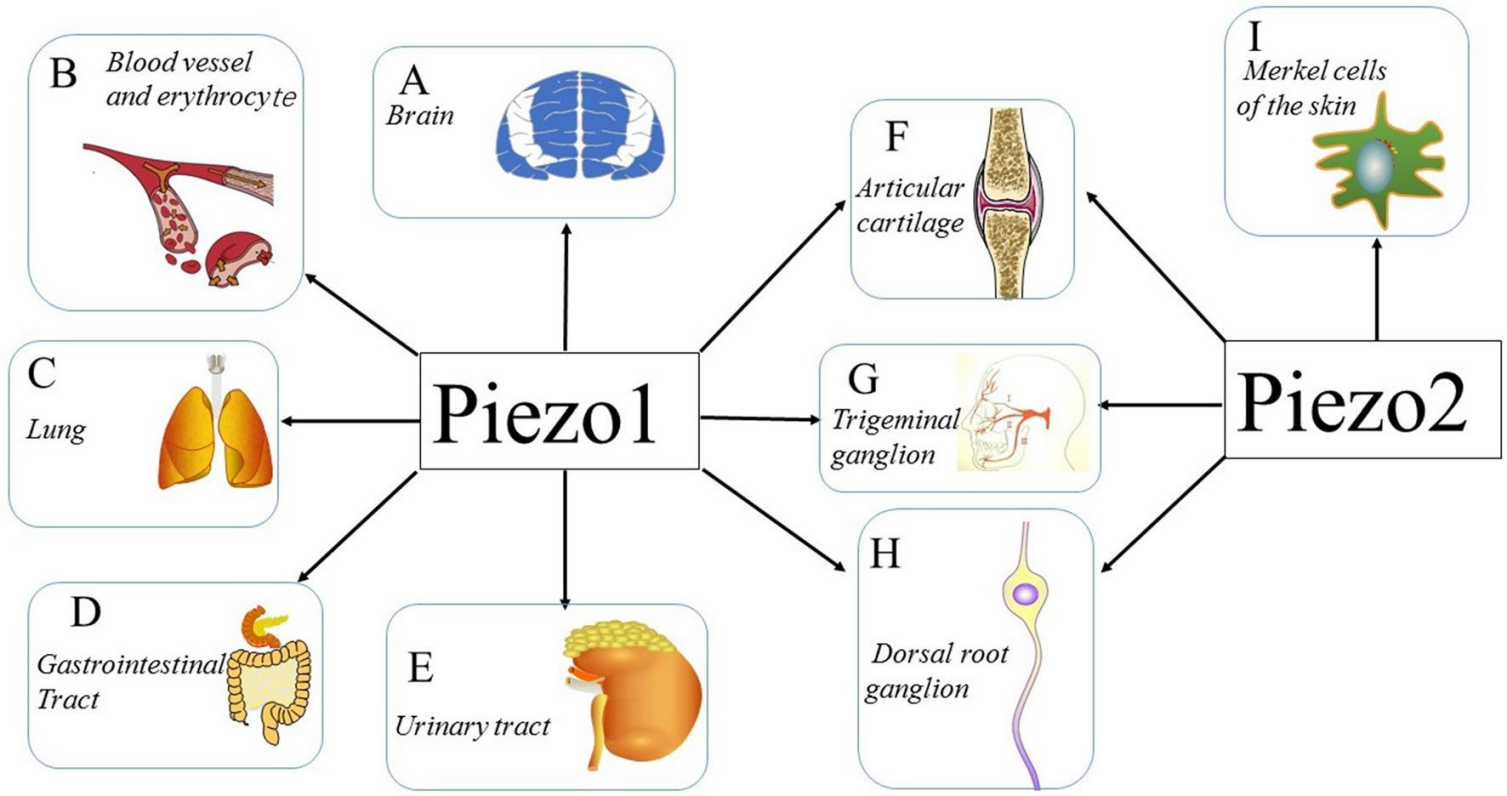
Expression and function of Piezo channels Multiple tissues and cells express Piezo channels, and each of those shown is discussed in this review. **a–e** demonstrate the vital role of the Piezo1 channel in the CNS, blood vessels, erythrocytes, lungs, gastrointestinal tract and urinary tract. **f–h** illustrate the expression of both the Piezo1 channel and Piezo2 channel in articular cartilage, trigeminal ganglia, and dorsal root ganglia. **i **shows that the Piezo2 channel is expressed in Merkel cells, which are involved in sensing light touch

Piezo channels are expressed in a wide range of mechanically sensitive cells and allow Ca^2+^ influx in response to different types of external forces, such as fluid flow [86], pulling [87], and ultrasonic forces [88]. The biological function of Piezo channels was recently investigated in a number of studies (Fig. 6). The results of these studies verified the pivotal roles of Piezo channels in mechanotransduction under physiological and pathophysiological conditions. Here, we focus on reviewing the biological function of Piezo channels in several different types of MS tissues and cells.

### Role of Piezo1 in endothelium morphogenesis and development

**Fig. 7:**
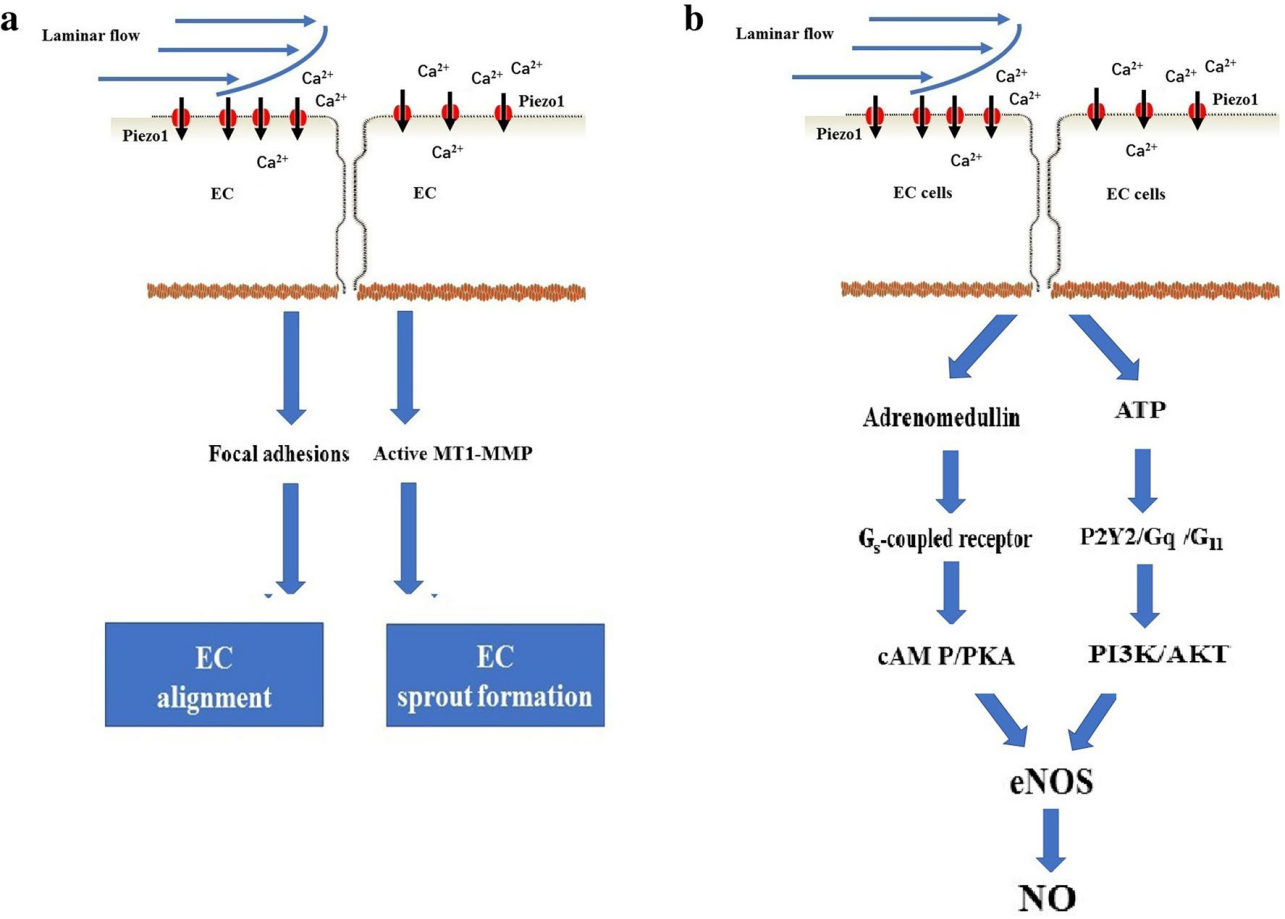
Role of the Piezo1 channel in vascular development and tone. **a** In blood vessels, shear stress (laminar flow: blue arrow) triggered Piezo1-mediated Ca^2+^ influx and thereby facilitated endothelial cell (EC) alignment via the regulation of focal adhesions and EC sprout formation via the activation of MT1-MMP signaling. **b** In blood vessels, shear stress (laminar flow: blue arrow) activated the Piezo1 channel in ECs and subsequently mediated vascular tone. Specifically, shear stress led to Piezo1-dependent adrenomedullin release in ECs, which then activated the G_s_-coupled endothelial adrenomedullin receptor. The subsequent increase in cAMP levels promoted the phosphorylation of endothelial NO synthase (eNOS) and caused NO production and vasodilation. Additionally, shear stress activated the Piezo1 channel in ECs and subsequently mediated the release of ATP in part by pannexin channels. Extracellular ATP, in turn, stimulated G_q_/G_11_-coupled purinergic P2Y2 receptors, resulting in the phosphorylation of eNOS via PI3K/AKT signaling and increased NO formation

Piezo1 channels are readily detected in a variety of endothelial cells (ECs), which are part of the vasculature, lymphatic vasculature and heart; these cells can directly sense physiological shear stress in the cardiovascular system [89]. Global and EC-specific disruption of Piezo1 in mice caused the mice to die in utero at mid-gestation due to defects in vascular formation [90, 91]. EC-specific Piezo1-knockout mice exhibited defective Ca2 + influx coupled with impaired EC alignment and remodeling of the cytoskeleton in response to wall shear stress [90] (Fig. 7a). The lymphatic ECs are the main component of the lymphatic valves whose formation is governed by Piezo1 channels [92]. In contrast, Piezo1-null humans have been reported to survive with generalized lymphatic dysplasia [63, 64]. The discrepancy between the two studies could be due to a compensatory mechanism in humans with homozygous mutations in the Piezo1 channel.

The Piezo1 channel is also required for vascular remodeling. Angiogenesis, the formation of new capillaries from existing vessels, is an essential feature of embryonic development, inflammation, wound healing, tissue repair, and tumor growth [93]. Kang et al. [94] showed that mechanical stimuli triggered Piezo1-mediated Ca2 + influx and thereby activated matrix metalloproteinase-2 and type 1-matrix metalloproteinase and synergistically facilitated sprouting angiogenesis (Fig. 7a). Additionally, disturbed flow led to integrin activation in a Piezo1- and Gq/G11-dependent manner, which caused focal adhesion kinase-dependent nuclear factor-κB activation [95]. EC-specific Piezo1-knockout mice exhibited reduced integrin activation, along with inflammatory signaling and atherosclerosis progression [95]. Taken together, these recently published papers have shown the considerable importance of the Piezo1 channel for EC alignment and migration, capillary network formation and endothelial inflammation.

### Role of Piezo1 in vascular tone

Vascular tone and blood pressure are primarily regulated by flow-induced vasorelaxation, which is mediated by vasodilator factors, such as nitric oxide (NO) [96–98]. Shear stress was shown to activate the Piezo1 channel in ECs and subsequently mediate the release of ATP, in part, through pannexin channels [99]. Extracellular ATP, in turn, stimulated G_q_/G_11_-coupled purinergic P2Y2 receptors and activated the shear sensing complex (PECAM-1/VE-cadherin/VEGFR), which resulted in the phosphorylation of endothelial NO synthase (eNOS) at Ser 1176 via PI3K/AKT and increased NO formation [99] (Fig. 7b). Furthermore, shear stress led to Piezo1-dependent adrenomedullin release in ECs, which then activated the G_s_-coupled endothelial adrenomedullin receptor [100]. The subsequent increase in cAMP levels promoted the phosphorylation of eNOS at Ser 633 through protein kinase A (PKA), causing NO production and vasodilation [100] (Fig. 7b). Similarly, the Piezo1 channel was found to be required for the vascular relaxation of the uterus [101] and intrapulmonary artery [102].

In contrast to its vasodilation effects, the Piezo1 channel appears to be involved in endothelium-dependent vasoconstriction in mesenteric arteries, which is closely related to peripheral resistance and blood pressure [103]. Mechanistically, Piezo1 channels in ECs oppose endothelium-dependent relaxation mediated by endothelium-derived hyperpolarization. Mice in which endothelial Piezo1 was disrupted had a normal blood pressure during inactivity but showed an elevated blood pressure during whole-body physical activity [103]. Intriguingly, these results appear to be contradict the finding that endothelial Piezo1-knockout mice exhibited an increase in mean arterial blood pressure at rest [99]. The inconsistency between the two studies could be due to differences in mouse genetic background.

Collectively, these studies suggest that the Piezo1 channel is involved in regulating vascular tone under pathological conditions but that the process is more complicated than our current understanding suggests. Thus, further studies are needed to elucidate the role of the Piezo 1 channel in vascular biology.

### Role of Piezo1/2 in the baroreceptor reflex

**Fig. 8:**
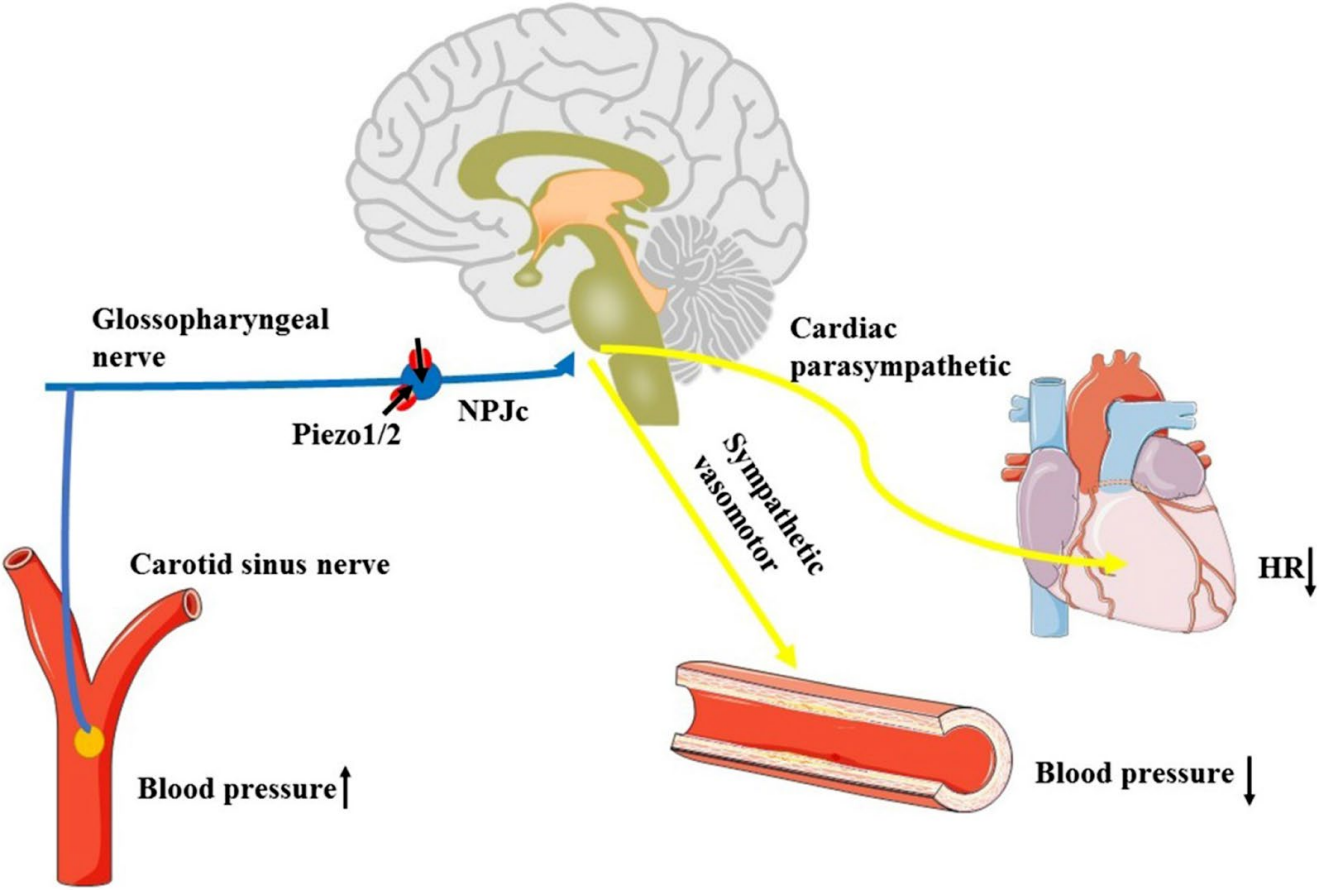
Overview of the Piezo1/2 channel-regulated baroreceptor reflex. Both Piezo1 and Piezo2 channels were highly expressed in the nodose-petrosal-jugular-ganglion complex (NPJc), which contains the cell bodies of baroreceptor neurons. Shear stress from high blood pressure was transformed into an electronic signal through Piezo1/2 channel activation, which was relayed to the medullary cardiovascular center. Subsequently, the efferent impulse contacted its effector organs (heart and blood vessels), decreasing the subject’s blood pressure and heart rate

Beat-to-beat short-term stabilization of blood pressure is often regulated by the baroreceptor reflex in which pressure sensors located primarily in the arterial walls of the carotid artery sinus and aortic arch rapidly respond to fluctuations in blood pressure [104, 105]. A recent publication by Zeng et al. [106] suggested that both Piezo1 and Piezo2 channels are highly expressed in the nodose-petrosal-jugular-ganglion complex (NPJc), which contains the cell bodies of baroreceptor neurons (Fig. 8). Conditional knockout of both Piezo1 and Piezo2 in the mouse NPJc fully impaired the baroreceptor reflex function and aortic depressor nerve activity, decreasing the heart rate and blood pressure, which is consistent with the clinical phenotype of patients with baroreflex failure. However, knockout of either Piezo1 or Piezo2 alone in this region failed to cause these changes. The activation of Piezo2-positive sensory afferents using optogenetic techniques was sufficient to trigger the baroreceptor reflex in mice (Fig. 8). Although recent reports have indicated that both Piezo1 and Piezo2 channels underlie baroreflex transduction, the molecular identity of this process remains a controversial issue due to other mechanoreceptors associated with the cardiovascular system.

**Fig. 9:**
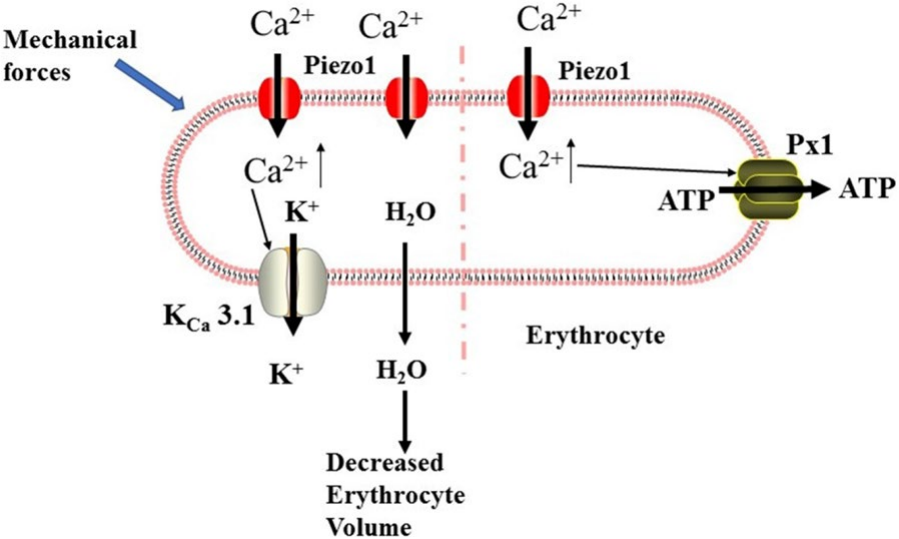
Role of Piezo1 in erythrocytes. The left side of the figure depicts the Piezo1 channel regulating erythrocyte volume. In overhydrated red blood cells (RBCs), Piezo1-mediated Ca^2+^ influx activates K^+^ efflux through the Gardos channel (KCa3.1), which in turn leads to water loss and RBC dehydration. The right side of the figure shows the working model for how Piezo1 channel activation regulates the release of ATP from erythrocytes. In erythrocytes, shear can activate the Piezo1 channel and induce Ca^2+^ influx, which triggers significant ATP release that is dependent on pannexin-1 (Px1)

### Role of Piezo1 in erythrocytes

Erythrocytes, the major blood constituents, have the capacity to undergo morphological deformations in response to various external forces in flow [107], which is critical for optimal functioning of erythrocyte and vascular physiopathology [108, 109]. Many Piezo1 mutations in humans have been linked to hereditary xerocytosis (HX), also called DHS, a dominantly inherited disorder of erythrocyte volume homeostasis [43, 50, 61, 110]. Furthermore, morpholino-mediated knockdown of the Piezo1 channel in zebrafish perturbed erythrocyte volume homeostasis [111]. These results implicated the potential role of Piezo1 in erythrocyte function.

Recently, it has been experimentally identified that the Piezo1 channel is involved in erythrocyte volume regulation via downstream activation of the KCa3.1 Gardos channel [39] (Fig. 9). In addition, a gain-of-function Piezo1-R2482H mouse model recapitulated most features of HX, providing equally compelling evidence for the essential role of the Piezo1 channel in erythrocyte function [112]. Interestingly, gain-of-function Piezo1 attenuated plasmodium infection in vitro, which is consistent with the phenomenon that erythrocyte dehydration includes HX protection against malaria [112]. Furthermore, Piezo1 has been shown to regulate shear-induced ATP release from human erythrocytes, a process that plays essential roles in vascular tone [113] (Fig. 9). However, despite these links between the Piezo1 channel and erythrocyte function, the effects of Piezo1 pharmacological inhibitors in HX patients have not been reported.

### Role of Piezo1 in the nervous system

Neurons in the vertebrate central nervous system (CNS) exhibit the ability to detect local mechanical signals that influence cell division, gene expression, cell migration, morphogenesis, cell adhesion, fluid homeostasis, ion channel gating and vesicular transport [114–116]. These mechanical signals originate primarily from the surrounding environment, such as from astrocytes and the ECM, as indicated by their structure and properties [117]. The link between the Piezo1 channel and the nervous system was first recognized because Piezo1 is positioned with a punctate distribution along the axons and growth cones of *Xenopus* retinal ganglion cells (RGCs), a part of the CNS [118]. Importantly, Piezo1 was also demonstrated to account for axon growth and regeneration [118]. Subsequently, the expression and distribution of Piezo channels in rodents were investigated. The Piezo1 channel was primarily located in myelinated axonal pathways of the mouse and rat brain, including in the corpus callosum and cerebellar arbor vitae, particularly in CNS neurons of the frontal cortex [119, 120]. In contrast, mature oligodendrocytes and astrocytes express much less Piezo1 protein [119]. Interestingly, for an unknown reason, peripheral infection and aging upregulate Piezo1 expression in reactive cortical astrocytes, consistent with a model of Alzheimer’s disease in aging rats [120]. Demyelinating diseases consist of disorders of the CNS that involve progressive degeneration of the myelin sheath, which is formed by specialized glial cells that normally surround neuronal axons [121]. Yoda1, a Piezo1 agonist, led to demyelination, while pharmacological inhibition of Piezo1 using GsMTx4 may prevent axonal and myelin damage in the CNS [119]. Together, these findings have shown that Piezo1 channels underlie CNS function.

**Fig. 10:**
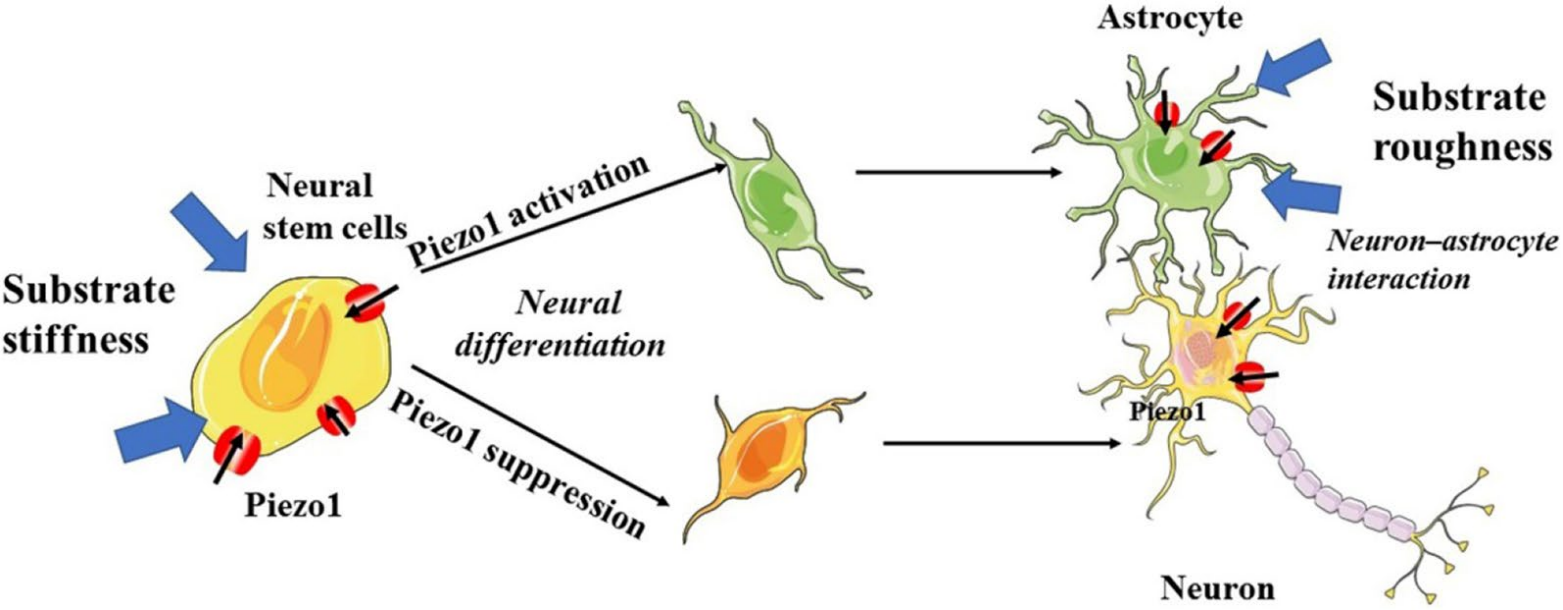
Role of the Piezo1 channel in regulating CNS processing. Mechanical properties of the neural progenitor cell environment activate Piezo1 channels in neural stem cells, astrocytes and neurons, thereby leading to neuronal differentiation, the development of neurite morphology and neuron–astrocyte interactions

Neural stem cells are self-renewing cells with the capacity to differentiate into neurons, astrocytes, and oligodendrocytes [122, 123]. This process is extremely sensitive to the mechanical properties of the cellular environment [124, 125]. The Piezo1 channel is expressed by human neural stem cells and plays an important role in directing the lineage choice of neural stem cells towards a neuronal or astrocytic phenotype [126] (Fig. 10). The activation of Piezo1 triggered by traction forces elicited Ca^2+^ influx and directed the lineage choice of neural stem cells towards a neuronal phenotype, while the inhibition or knockdown of Piezo1 suppressed neurogenesis and enhanced astrogenesis [126]. These findings introduce an intriguing question: Is the Piezo1 channel also involved in astrocyte-neuron interactions that are key for the maintenance and regulation of neuronal function? An elegant study by Blumenthal et al. [127] showed that pharmacological inhibition of Piezo1 abolished neuronal sensitivity to nanoroughness, a mechanical signal resulting from neighboring cells and ECM molecules, and sequentially promoted the decoupling of neurons from astrocytes, thus providing evidence for the role of Piezo1 in neuron–astrocyte interactions. This information provides a clue for answering this question **(**Fig. 10).

### Role of Piezo1/2 in the gastrointestinal (GI) tract

The vast majority of the body’s serotonin (5-hydroxytryptamine; 5-HT), an important paracrine and neurotransmitter molecule in the gut, is synthesized by gastrointestinal epithelial enterochromaffin cells in response to mechanical force within milliseconds [128]. Wang et al. [129] discovered that the Piezo2 channel is highly and specifically enriched in human and mouse enterochromaffin cells and that it contributes to 5-HT release. In the stomach, G cells are a particularly important population of enteroendocrine cells that produce gastrin to control gastric activities and are present almost exclusively in the antral gastric mucosa [130, 131]. Lang et al. [132] further showed that the vast majority of G cells in the gastric mucosa of mice expressed the Piezo1 channel, indicating that the Piezo1 channel might play an important role in the regulation of gastrin secretion. The role of Piezo1 channel activation in gastrin secretion requires further clarification.

The enteric nervous system includes both the submucosal plexus and the myenteric plexus and is essential for the autonomous regulation of regional secretory and absorptive functions in the GI [133]. Multifunctional MS enteric neurons have been identified [134, 135]. While the expression of Piezo2 is extremely rare in the somata of enteric neurons and Piezo2 is present in few neurites, Piezo1 is expressed by both enteric neuronal cell bodies and fibers in the myenteric and submucosal plexi of guinea pigs, mice and humans [136]. Surprisingly, an activator and inhibitor of Piezo channels had no effect on the mechanotransduction process of MS enteric neurons [136], which may be in part due to the off-target effects of the pharmacological activator and inhibitor. Further work is required to determine the direct link between Piezo channel activation and the mechanosensitivity of enteric neurons.

### Role of Piezo1/2 in joints

Articular cartilage, in which chondrocytes are the only cell type, is a load-bearing tissue that facilitates joint articulation and minimizes friction in diarthrodial joints [137]. The significance of mechanical loading in regulating chondrocyte anabolic and biosynthetic activity has been well documented [138]. A link between the Piezo2 channel and joint characteristics has been implied by the presence of mutations in Piezo2 in several arthrogryposis disorders [66–68]. Furthermore, robust expression of both Piezo1 and Piezo2 has been detected in isolated primary chondrocytes and in the knee joint cartilage [139]. Specifically, when Piezo1 or Piezo2 channels were individually knocked down in chondrocytes, the MA Ca^2+^ influx was virtually eliminated [139]. This result indicates that Piezo1 and Piezo2 together contribute to MA Ca^2+^ signaling in chondrocytes. Additionally, the inhibition of Piezo1 and Piezo2 eliminated the mechanical response in primary articular chondrocytes and reduced chondrocyte death after mechanical injury in vivo, suggesting the vital role of Piezo channels in cartilage mechanotransduction upon injury [139]. However, the question of how Piezo1 and Piezo2 channels cooperate to function synergistically at the molecular level remains unclear.

### Role of the Piezo 1 channel in lung diseases

**Fig. 11:**
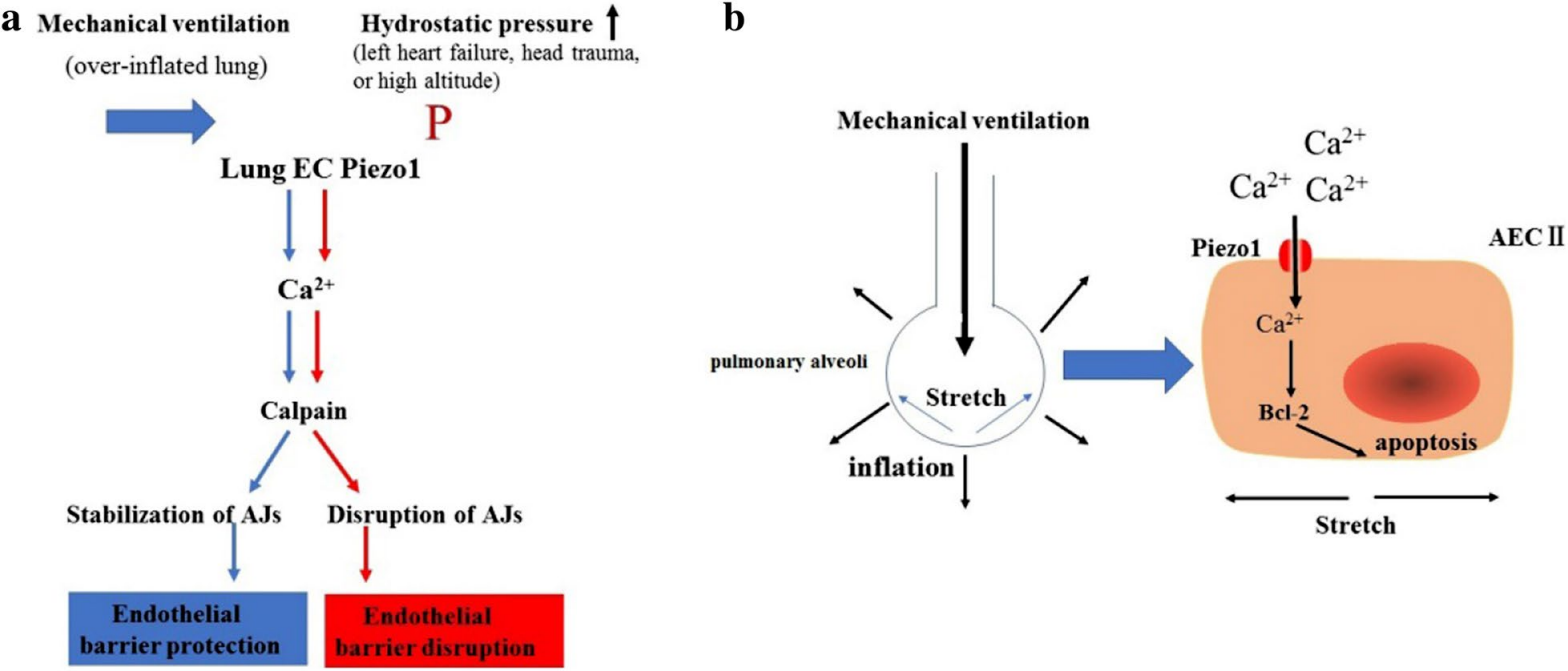
Role of the Piezo1 channel in lung vascular endothelial cells (ECs) and alveolar type II epithelial cells (AEC-IIs). **a** Shear stress (mechanical ventilation: blue arrow) triggered Piezo1-mediated Ca^2+^ influx and thereby stabilized adherens junctions (AJs) of ECs through calpain. Piezo1 activation by hydrostatic pressure (P, red arrow) in lung ECs caused disruption of the AJs. **b** Alveoli exposed to cyclic stretch due to mechanical ventilation. Mechanical stretching of the alveoli induced Piezo1 channel activation of AEC-IIs, which resulted in the apoptosis of AEC-IIs via Ca^2+^ influx

As observed in the vascular endothelium, the Piezo1 channel is highly expressed in the lung endothelium, which is exposed to mechanical forces due to increases in alveolar pressure and hydrostatic pressure [49, 13, 40]. Increased hydrostatic pressure in pulmonary capillaries, a characteristic of left heart failure, head trauma, or high altitude, activated Piezo1 and induced lung vascular hyperpermeability by promoting degradation of the endothelial adherens junction proteins VE-cadherin, β-catenin, and p120-catenin [141] (Fig. 11a). Nevertheless, another study found that the Piezo1 channel helped to improve the lung endothelial barrier function and alleviated ventilator-induced lung injury (VILI) caused by over-inflated lung [142]. Piezo1 activation in lung ECs prevented lung endothelial barrier breakdown in response to alveolar stretch by suppressing Src-induced VE-cadherin phosphorylation [142] (Fig. 11a). The reason for the contradictory results between these two studies is unclear_。_.

The lung tissue epithelium and the surrounding tissues undergo mechanical stretch generated by air under physiological conditions and mechanical ventilation [143]. A recent study [144] demonstrated that the Piezo1 channel was highly expressed in alveolar epithelial type ii cells (AECs), a component of the alveolar epithelium. Over-inflated alveolae caused by mechanical stretch during mechanical ventilation activated the Piezo1 channel, resulting in Ca2 + influx. Subsequently, the elevated intracellular Ca2 + signal led to the apoptosis of AECs, probably through the Bcl-2 pathway [144] (Fig. 11B). Additionally, mechanical stretch activated Piezo1 channels in alveolar type I cells to trigger ATP release and paracrine stimulation of surfactant secretion from alveolar type II cells, which is essential to maintain lung function [145].

Given the importance of the Piezo1 channel in lung disease, it is reasonable to ask whether mammalian Piezo1 plays a role in other lung diseases, such as chronic obstructive pulmonary disease, asthma, and mechanical stretch-induced pulmonary fibrosis. Clearly, further studies are warranted to better understand the function of Piezo1 in lung disease.

### Role of Piezo2 channel in Hering–Breuer inflation reflex

**Fig. 12:**
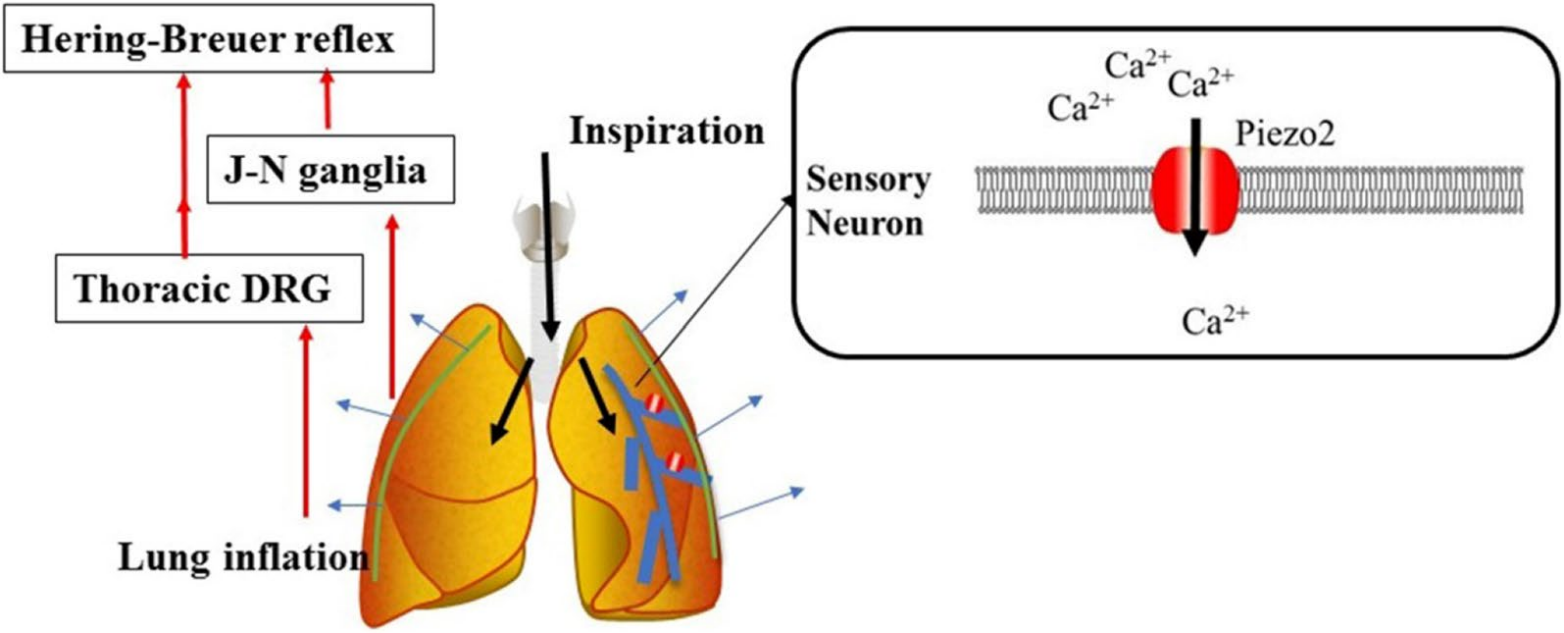
Overview of the Piezo2-regulated Hering-Breuer inflation reflex. Piezo2 channels are expressed in airway vagal sensory neurons. Shear stress from lung inflation triggers the Piezo2 channel and activates the Hering-Breuer reflex through the jugular-nodose ganglia complex and the thoracic dorsal root ganglia

Vagal nerve treatment of the Hering-Breuer inflation reflex ended the inspiratory phase and produced a prolonged expiratory phase; these alterations played a role in the regulation of physiological respiratory function [146]. A direct link between the Piezo2 channel and the Hering-Breuer inflation reflex was shown in global and tissue-specific Piezo2-deficient mice [147]. Nonomura and colleagues found that both the global and sensory neuron-specific deletion of Piezo2 led to respiratory distress and death in new-born mice that did not have impaired embryonic lung development [147]. Vagal neuron-specific Piezo2-deficient mice survived to adulthood, but the Hering-Breuer reflex in these mice was impaired, as reflected by increased tidal volume and prolonged expiratory airflow. Strikingly, optogenetic stimulation of both Piezo2 and vagal neurons in adult mice resulted in the cessation of respiration with a decrease in the average breathing rate (Fig. 12). Interestingly, gain-of-function mutations in Piezo2 and Piezo2 deficiency in humans have been associated with respiratory diseases, including respiratory insufficiency at birth, chronic obstructive pulmonary disease, and sleep apnea in adults [66–68, 148, 149]. These results indicate that Piezo2 might be required for respiration in new-borns and to regulate normal breathing in adults.

### Role of Piezo channels in the urinary tract

Of critical importance to the proper function of the organs that comprise the urinary tract (kidneys, ureters, bladder, and urethra) is the ability to sense and respond to changes in both fluid flow and intraluminal pressure [150]. It was recently shown that Piezo1 is expressed throughout the urinary tract, including in epithelial cells, interstitial cells, and smooth and striated muscle cells [151]. One study showed that renal epithelial cell Piezo1-deficient mice at the adult stage had an altered urinary concentration following dehydration or fasting [152]. Additionally, Piezo1 is required for stretch-activated ion channel activity in proximal convoluted tubule cells, but its stretch sensitivity is highly regulated by polycystin-2 [153]. The Piezo1 channel is also present in the mouse and human urothelium and plays a functional role in the physiological function of the bladder [154]. Primary urothelial cells isolated from bladders exhibited Piezo1-dependent Ca^2+^ influx in response to mechanical stretch stimuli, leading to potent ATP release; this response, however, was suppressed when cells were treated with Piezo1-targeted siRNAs or GsMTx4, a pharmacological inhibitor of Piezo1 [151]. In addition, the expression levels of Piezo1 in the bladder were significantly increased in bladder dysfunctions, including in partial bladder outlet obstruction and chronic cystitis-associated bladder hyperactivity [155]. Overall, the Piezo1 channel is widely expressed in the urinary tract and has also been shown to be required for the physiological function of urinary systems.

### Role of Piezo channels in touch, proprioception and pain

The fundamental senses, including touch, proprioception, and mechanical pain, rely on DRG pseudounipolar neurons connecting the skin, muscle, and internal organs with the spinal cord through the central and peripheral axonal branches [156, 157]. Studies have found that the Pizeo2 channel is abundantly expressed in a subset of sensory DRG neurons, including in cutaneous mechanoreceptors, known as Merkel cell-neurite complexes [33, 158]. *Drosophila melanogaster* Piezo channels and zebrafish Piezo2b channels were shown to be involved in somatosensory mechanotransduction [159, 160]. Furthermore, mice with Piezo2-conditional deletion in the sensory neurons or in Merkel cells displayed severe deficits in response to gentle touch, but the mechanical nociception sensitivity depending primarily on Aδ and C fibers was unaffected in Piezo2-deficient mice. This provides definitive evidence of the involvement of the Piezo2 channel in touch [158, 161, 162] (Fig. 6). The Piezo2 channel was found to be present in the sensory endings of proprioceptors, providing the body with the information needed to produce coordinated movement. The critical role of the Piezo2 channel in mediating proprioception was revealed using proprioceptive neuron-specific conditional Piezo2-deleted mice [163]. Together, these studies suggest that the Piezo2 channel is required not only for touch sensation mediated by Aβ fibers but also for proprioception.

Tactile pain, or mechanical allodynia, is pain generated by innocuous tactile stimuli via primary afferent sensory neurons. Recently, two elegant studies have shown that Piezo2 channel knockout completely abolished tactile allodynia in mice [164, 165], whereas mechanical nociception was only partially dependent on this ion channel. This is consistent with the view that inflammatory signals enhance Piezo2-mediated MS currents in vitro and induce mechanical hyperalgesia. In particular, individuals with autosomal recessive inheritance of loss-of-function mutations in Piezo2 display major deficits in discriminative touch perception and coordinated movement production, as well as painful reactions to touch after skin inflammation, implying that the Piezo2 is indispensable for sensing light touch, proprioception, and tactile allodynia in humans [164]. Interestingly, a recent study [166] reported that Piezo2 deletion not only impaired touch but also surprisingly increased mechanical nociception, which is consistent with the idea that touch normally suppresses pain [167, 168]. However, another study showed contradictory results regarding the presence of Piezo channels in DRG neurons, showing the selective expression of Piezo1 in small-diameter DRG neurons [169]. Since smaller DRG neurons are key for the sensation of acute noxious stimuli, these findings directly implicate the involvement of Piezo1 in mechano-nociception.

Trigeminal ganglia innervate head and face tissues and are implicated in the generation of migraine pain. Recent studies [170] found the expression of both Piezo 1 and Piezo2 channels in trigeminal sensory neurons, which suggests their potential roles in migraine-related mechanotransduction. The application of Yoda1 to the extended receptive field of meningeal afferents induced the massive and prolonged activation of trigeminal nerve fibers [170]. Moreover, Yoda1 stimulation activated trigeminal neurons and triggered the release of calcitonin gene-related peptide (CGRP), the main migraine mediator, which is known as a powerful promoter of meningeal inflammation and trigeminal neuron sensitization.

Although current research suggests that Piezo channels are closely related to the transmission of touch sensation, proprioception and pain, the role of the Piezo1 subtype in these processes, particularly in mechanical nociception, is unclear. Further investigations are needed to elucidate the mechanical sensitivity of Piezo1 channels in response to noxious mechanical stimuli.

### Role of Piezo channels in tumorigenesis

Tumor cells are exposed to extracellular environments with mechanical stimuli, such as tissue pressure (stiffness), cell membrane tension and ECM components. Clinically, matrix stiffening is a prominent hallmark of the tumor microenvironment (TME). As tumor cells grow, the mechanical forces within the tumor and TME increase, and the mechanosensitive cation channels are activated by these mechanical signals and interact with focal adhesions to regulate tumor development [171]. Recent research showed that the Piezo1 channel is closely correlated with some types of cancers, including oral squamous cell carcinoma, prostate cancer and colon cancer [172–174]. Furthermore, the Piezo2 channel has been shown to play a role in the proliferative changes and angiogenesis of brain metastatic cells and bladder cancer [175]. Interestingly, Piezo1 also links physical forces to immune regulation in myeloid cells, and these cells regulate cancer and infectious disease [176]. This suggest that Piezo channels have a crucial role in tumor development and may, therefore, be a novel therapeutic target for cancer treatment.

## Summary and outlook

Without question, a more in-depth understanding of the structural details, kinetic properties and structure-function relationships of Piezo channels foreshadows a new age of research on the pathological and physiological processes associated with mechanotransduction. Nevertheless, many new questions have been raised, and new perspectives have begun to emerge. For example, whether Piezo2 and Piezo1 possess similar structures and mechano-gating mechanisms remains unknown. Do posttranslational modifications of Piezo channels regulate their activation? Would targeting Piezo channels be an effective therapeutic approach for the treatment of human diseases associated with Piezo channel mutations? The manner in which Piezo channels stabilize their open conformation is also unknown. Future research efforts to more fully understand the structure-function relationship of Piezo channels should also be oriented towards obtaining the structures of Piezo channels in different conformational states, and a more in-depth investigation of the structure-guided functional characterization should be carried out.
